# Direct Reflectance Measurements from Drones: Sensor Absolute Radiometric Calibration and System Tests for Forest Reflectance Characterization

**DOI:** 10.3390/s18051417

**Published:** 2018-05-03

**Authors:** Teemu Hakala, Lauri Markelin, Eija Honkavaara, Barry Scott, Theo Theocharous, Olli Nevalainen, Roope Näsi, Juha Suomalainen, Niko Viljanen, Claire Greenwell, Nigel Fox

**Affiliations:** 1 Finnish Geospatial Research Institute FGI, National Land Survey of Finland, Geodeetinrinne 2, 02430 Masala, Finland; teemu.hakala@nls.fi (T.H.); eija.honkavaara@nls.fi (E.H.); olli.nevalainen@nls.fi (O.N.); roope.nasi@nls.fi (R.N.); juha.suomalainen@nls.fi (J.S.); niko.viljanen@nls.fi (N.V.); 2 National Physical Laboratory NPL, Teddington TW11-0LW, UK; barry.scott@npl.co.uk (B.S.); theo.theocharous@npl.co.uk (T.T.); claire.greenwell@npl.co.uk (C.G.); nigel.fox@npl.co.uk (N.F.)

**Keywords:** radiometric calibration, spectral calibration, imaging spectrometer, radiance, reflectance, drone, UAV, hyperspectral

## Abstract

Drone-based remote sensing has evolved rapidly in recent years. Miniaturized hyperspectral imaging sensors are becoming more common as they provide more abundant information of the object compared to traditional cameras. Reflectance is a physically defined object property and therefore often preferred output of the remote sensing data capture to be used in the further processes. Absolute calibration of the sensor provides a possibility for physical modelling of the imaging process and enables efficient procedures for reflectance correction. Our objective is to develop a method for direct reflectance measurements for drone-based remote sensing. It is based on an imaging spectrometer and irradiance spectrometer. This approach is highly attractive for many practical applications as it does not require in situ reflectance panels for converting the sensor radiance to ground reflectance factors. We performed SI-traceable spectral and radiance calibration of a tuneable Fabry-Pérot Interferometer -based (FPI) hyperspectral camera at the National Physical Laboratory NPL (Teddington, UK). The camera represents novel technology by collecting 2D format hyperspectral image cubes using time sequential spectral scanning principle. The radiance accuracy of different channels varied between ±4% when evaluated using independent test data, and linearity of the camera response was on average 0.9994. The spectral response calibration showed side peaks on several channels that were due to the multiple orders of interference of the FPI. The drone-based direct reflectance measurement system showed promising results with imagery collected over Wytham Forest (Oxford, UK).

## 1. Introduction

Remote sensing based on Unmanned Aerial Vehicles (UAVs, drones) has evolved rapidly in recent years. Miniaturized hyperspectral sensors operable from drones have entered the market, providing 2D format snapshot imaging, and offering new possibilities for acquiring information on our environment and sustainable use of natural resources [1,2,3,4].

Reflectance is a physically defined object property and therefore often preferred output of the remote sensing data capture to be used in the further processes [5]. The prerequisite for any reflectance factor processing is production images in radiance or pseudoradiance units [6]. Processing raw images to radiances include steps such as dark current subtraction and flat field correction. Additional corrections for chromatic aberrations, filter transmittance effects, and spectral smile are often necessary for hyperspectral sensors [7,8]. Previously, for example Büttner and Röser [9] calibrated a line scanner UAV system using an integrating sphere with traceable procedures. Radiometric calibration of spectral instruments is typically carried out in laboratory using integrating spheres or by illuminating reference panel with calibrated lamp [10,11,12,13,14], and spectral calibration is performed using monochromator or line emission lamps [10,11,12,15].

When accurately calibrated, frame format hyperspectral cameras can enable quantitative measurements and sensitive application of sensor, as they can produce 3D hyperspectral features with very high spatial resolutions, such as 1–5 cm. However, these cameras also have challenging issues in calibration, including stability, dark signal non-uniformity (DSNU), shape and cleanness of spectral response, spectral smile, keystone, etc. Aasen et al. [1] and Yang et al. [16] calibrated the Cubert UHD Firefly snapshot hyperspectral camera, and they faced several challenges in the calibration process, including temperature dependency on sensor dark current. Flat-field analysis by Aasen et al. [1] revealed a clear decrease of the DN values towards the edges of the image and an additional undulated pattern causing significant challenges for calibration. Additionally, the lab calibration failed because the mechanical stability of the camera was not good enough. Also efficiency fall-off of the detector towards its spectral sensitivity borders and the decreased intensity towards the edges of the image reduced the precision of the radiometric information. The results by Yang et al. [16] indicated that the CCD of the hyperspectral sensor exhibited a noticeable vignetting effect and strips. These results indicated that the radiometric and spectral calibration of these systems is of great importance.

To process the radiance images to reflectance factors, methods using reflectance panels are often feasible because the UAV pilot is operating the system locally in the mapping area. At simplest, single highly reflective target has been used by taking calibration image on ground before or after the remote sensing flight [1,16,17], however, this approach is not considered as recommended due to many potential error sources [18]. When two or more reflectance targets are available on ground, and assuming that the illumination conditions in targets and in the object being studied are similar, empirical line based approaches can be used [6,19,20]. These conditions are not always met in UAV campaigns. For example, the UAV campaigns are often carried out in changing illumination conditions with varying cloudiness [21,22,23,24]; in such case the in situ reflectance panels do not offer a suitable solution. Secondly, in many situations it is also impossible to reliably employ reflectance panels on the ground at the campaign area. For example, in forests the illumination conditions are completely different on ground and at treetops, which are most often the target of interest [22]. Additionally, panels might not be suitable when operating beyond line of sight.

Recent studies have proposed various approaches to compensate for the challenges caused by varying illumination conditions during campaigns. The most promising approaches are an image based block adjustment [2,4,25] and correction based on measurement of illumination changes with irradiance sensor on ground or on-board UAV [21,23,24,26,27]. In the cases when the only changing component during the flight is the sun elevation, linear interpolation of the calibration parameters between the start and end of the campaign has been suggested [9,17]. Challenge with irradiance sensors on board drone is the requirement for aligning the irradiance spectrometer accurately in vertical direction [23]. As the irradiance measurement uses cosine weighed optics, the reading is highly dependent on the angle between the cosine collector and vertical vector. For example, if the solar elevation is 45°, deviation of one degree in the angle of the cosine collector will cause an error of ~2% to the irradiance value. As the UAV can easily be tilted ±5° or more, this is a major error source. The effect can be mitigated or even eliminated using an actively stabilized platform for the irradiance sensor.

Our objective in this study was to implement a drone system for direct reflectance measurement and to study its’ performance in forested environment. The proposed system does not require in situ reflectance panels for converting the sensor radiances to ground reflectance factors, in order to simplify the drone based remote sensing process. The major components of the system are an imaging 2D format hyperspectral camera and an irradiance spectrometer both installed on board drone. A calibration procedure was designed to perform the system calibration. The radiometric and spectral calibration of the 2D format hyperspectral camera was carried out using procedures having a metrological traceability in the National Physical Laboratory (NPL, Teddington, UK), focusing the spectral response and absolute calibration. Calibration of the irradiance spectrometers were based on the NIST traceable procedures of sensor manufacturers. A flight campaign was carried out using the novel imaging spectrometer system in a forest environment.

We describe the overall system and its components, the system calibration and datasets in Section 2 and Section 3. Results are given in Section 4 and discussed in Section 5.

## 2. Materials and methods

### 2.1. Proposed Method for Direct Hyperspectral Reflectance Measurement from Drone

We propose a drone based remote sensing system for measuring the reflectance with a 2D frame format hyperspectral camera measuring the reflected radiance and an integrated downwelling irradiance spectrometer. The reflectance factor measurement is based on the concept of bidirectional reflectance factor (BRF) $R\left(\theta_{i},\varphi_{i},\theta_{r},\varphi_{r},\lambda \right)$ [5,28]:

(1) $$R\left(\theta_{i},\varphi_{i},\theta_{r},\varphi_{r},\lambda \right)=\pi \frac{dL_{r}\left(\theta_{i},\varphi_{i},\theta_{r},\varphi_{r},\lambda \right)}{dE_{i}\left(\theta_{i},\varphi_{i},\lambda \right)}\;,$$

where $L_{r}\left(\theta_{i},\varphi_{i},\theta_{r},\varphi_{r},\lambda \right)$ is the radiance reflected by the object, $E_{i}\left(\theta_{i},\varphi_{i},\lambda \right)$ is the irradiance at object, $\theta_{i}$ and $\varphi_{i}$ are the zenith and azimuth angles of incident light, $\theta_{r}$ and $\varphi_{r}$ are the zenith and azimuth angles of reflected light.

**Figure 1:**
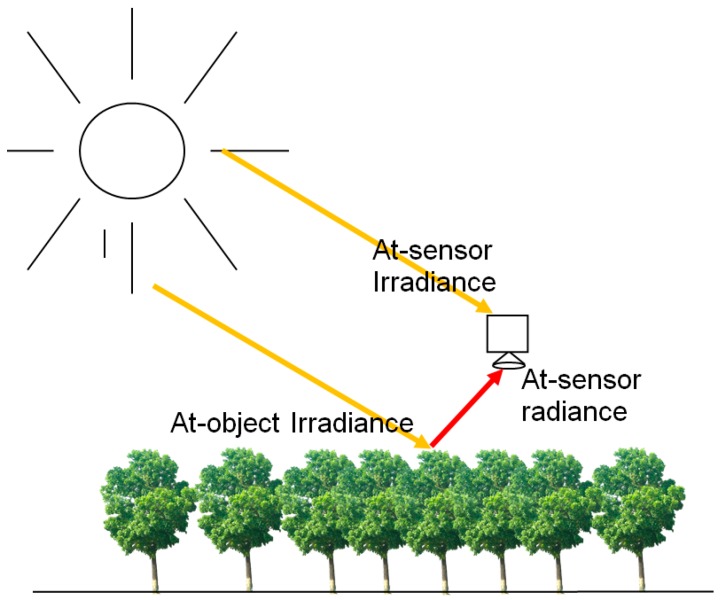
Principle of the direct reflectance measurement from drone.

In order to carry out the radiometric measurements, the irradiance level at the object and the radiance reflected by the object have to be measured. In the proposed approach, the downwelling irradiance is measured using an irradiance spectrometer, while the object radiance is measured using a hyperspectral camera. The principle is illustrated in Figure 1. It is critical to measure irradiance while the sensor is pointing directly upwards, as tilting the irradiance sensor only a few degrees results drastic changes in recorded irradiance. This means that the irradiance sensor has to be stabilized. In this study, as the gimbal stabilization of the irradiance sensor on board UAV did not work, we developed an approach where we used a reference irradiance sensor on ground to level the UAV-born irradiance data. The system setup at the Finnish Geospatial Research Institute (FGI, Masala, Finland) and the calibration activities are described in the following sections.

### 2.2. UAV Based 3D Imaging Spectrometer System

#### 2.2.1. The 2D Frame Format Hyperspectral Camera

A 2D format hyperspectral camera, based on tuneable FPI-filter [3,29] is used as the main sensor. The FPI camera prototype 2012b captures hyperspectral images in the range of 500–900 nm with 10–30 nm full width at half maximum (FWHM) [2,29]. The FPI camera has a focal length of 10.9 mm, aperture of f/2.8, and uses a CMOSIS CMV4000 RGB image sensor. The image size is 1024 × 648 pixels with a pixel size of 11 μm. The spectral range of light reaching the sensor is controlled by adjusting the air gap of the Fabry-Pérot interferometer filter. The sensor is a Bayer pattern RGB sensor allowing obtaining up to three wavelength bands on single exposure while utilizing multiple interferences. The interval between adjacent exposures is 0.075 s, and a full raw data cube consisting of 24 exposures can be acquired in 1.8 s. The number of resulting bands depends on the selected sequence of air gaps. The sequence used in this study provided 36 different bands. The field of view (FOV) is ±18° in the flight direction, ±27° in the cross-flight direction, and ±31° from corner to corner. The entire camera system weighs less than 700 g.

The camera manufacturer has calibrated the camera and provided the software for post processing of the raw FPI data to pseudoradiance images. The dark current correction is performed by subtracting from the captured images a dark image taken before or after the flight with covered lens. The photo response non-uniformity correction (PRNU) is determined in laboratory using a flat-field based on an integrating sphere; the PRNU correction includes the corrections for light falloff and the CMOS sensor non-uniformity [3]. The manufacturer has determined the absolute radiance calibration of the camera using an integrating sphere with two output ports. During the calibration, the FPI camera was installed in one of the ports and the radiance of the sphere was observed from another port using the ASD Field Spec Pro spectrometer (ASD, Analytical Spectral Devices, Boulder, CO, USA) belonging to the FGI. The manufacturer post-processing software gives so-called pseudoradiance images as outputs. Finally the integration time is taken into account to convert pseudoradiance to radiance in units W/(m^2^ sr nm). These radiance images are the starting point in further analysis in this study, and the radiance based on sensor manufacturer’s process is called *L_Man_*.

#### 2.2.2. Irradiance Spectrometers

Hemispherical spectral irradiance was measured with two spectrometers during the flight. Firstly, a small Ocean Optics USB2000+ spectrometer (OO, Ocean Optics, Largo, FL, USA) with spectral range of 350–1000 nm measured irradiance on board the UAV. Secondly, the aforementioned ASD spectrometer with a spectral range of 350–2500 nm measured stationary irradiance on ground with 5 s intervals. Both spectrometers were equipped with cosine corrected diffusing optics and each spectrum had a GPS timestamp for time synchronization with other measurements. The ASD was also used as reference radiometer for measuring point-wise spectral radiance of reference targets.

Radiometric calibration for the ASD spectrometer was performed at Analytical Spectral Devices calibration facility in April 2015. Calibrations at ASD Inc. were performed using verifiable NIST-traceable irradiance, reflectance and wavelength standards. According to the calibration document, the accuracy of calibration is better than 3% in wavelength range of the FPI camera (500–900 nm). The OO was calibrated at FGI using a tungsten-halogen calibration lamp HL-2000-CAL (Ocean Optics, Largo, FL, USA). Radiometric calibration for the lamp is provided by the manufacturer using a NIST traceable procedure.

### 2.3. Laboratory Calibration of the FPI Camera

#### 2.3.1. Spectral Calibration

**Figure 2:**
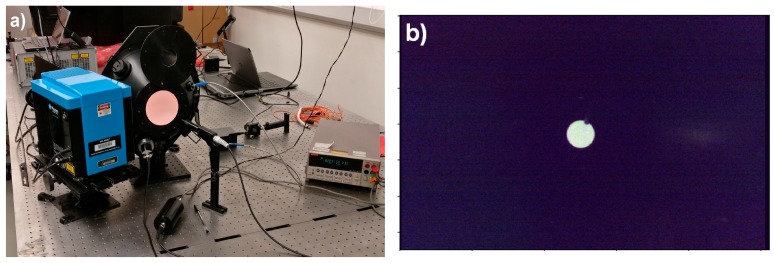
(**a**) The measurement setup for spectral response characterisation. Broadband light from a supercontinuum laser source (background) is directed to a monochromator (left) via an optical fibre. Resultant monochromatic light is fed to an integrating sphere, where a photodiode is used to monitor the light intensity. ASD FieldSpec pro is also used to monitor the output port (the optics and optical fibre visible in front of the sphere) (**b**) A FPI image of the integrating sphere port acquired in the calibration.

Spectral response calibration of the FPI camera was performed at the National Physical Laboratory using supercontinuum laser source (NKT Photonics UK, formerly Fianium, Southampton, UK) that was connected to a computer controlled tuneable monochromator (PhotonEtc, Laser Line Tuneable Filter, Montreal, QC, Canada). The output from the monochromator was connected to an integrating sphere. The light level of the sphere was monitored using a calibrated photodiode, and additionally the ASD was set to monitor the output port of the integrating sphere (Figure 2). The FPI camera was fixed to a distance of 2 m from the output port in order to keep the port in focus.

The FWHM of the monochromator was 1.0 nm, and relative wavelength resolution FWHM/8. Due to laboratory time limitations, wavelength was incremented in 1 nm steps in four selected essential spectral regions of the FPI camera (green, red, red-edge, NIR), and in 2 nm and 4 nm steps elsewhere to cover full spectral range of the camera. The uncertainty of the sphere radiance was ±0.44% in the wavelength range of the FPI camera.

The spectral response calibration was performed by acquiring five images of the output port of the integrating sphere with the FPI camera, and simultaneously recording the reading of the calibrated photodiode, and acquiring an ASD spectrum. First a set of measurements were taken from unilluminated sphere, followed by a new set of measurements from illuminated sphere. After this the centre wavelength of the input light was sifted by 1–4 nm and the measurements were repeated.

From the FPI pseudoradiance images the pixels covering the output port of the sphere were selected and only these pixels were used in further processing steps. The five sequential images of same target were averaged to a single value for each FPI camera channel. The unilluminated image values were then removed from the following illuminated values, correcting also impacts of the temperature change of the FPI camera. These values were then divided by the photodiode values.

The position of the peak wavelength of each channel was acquired by fitting a Gaussian shape to a window of ±50 nm around the maximum value. The Gaussian fitting was done with PeakFit -function from O’Haver [30]. The central peak wavelength and the FWHM of the Gaussian were then used as nominal values to describe the FPI channels. However, due to non-Gaussian response characteristics, the raw spectral response data acquired in this step were used to integrate NPL reference radiance (Section 2.3.2) to match FPI sensor response.

#### 2.3.2. Absolute Radiance Calibration

**Figure 3:**
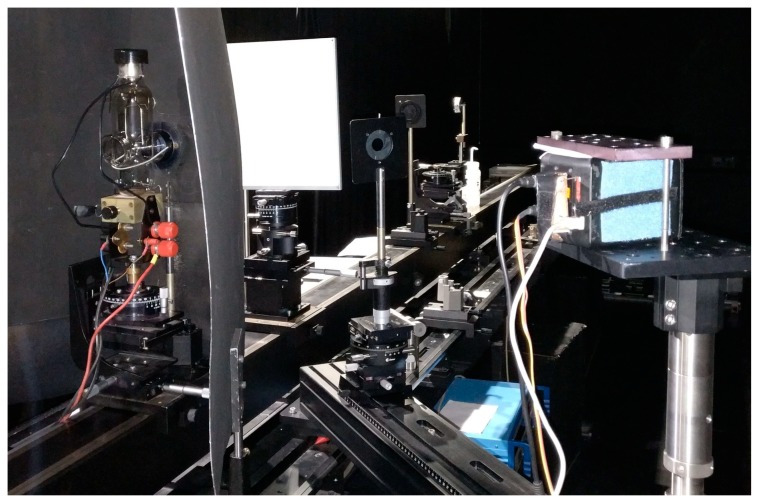
The radiance calibration setup. The lamp is placed on an optical rail to a known distance from a reflectance panel. The FPI camera is viewing the panel from 45° angle. Stray light is blocked by light baffles (additional baffles were placed after taking this image).

The absolute radiance calibration was accomplished by illuminating a reference panel using a lamp with calibrated irradiance (*E*) and acquiring images of the illuminated panel using the FPI camera. The reflectance factor (*R*) of the panel and distance between the lamp and the panel were also measured and thus the radiance of the illuminated panel could be calculated. The FPI camera was placed to an angle of 45° from the panel at a distance of 1 m (Figure 3). Two lamps were used: a FEL lamp and a Polaron lamp, both of them were calibrated at NPL for irradiance level at 500 mm distance from the lamp. With the Polaron lamp measurements were taken from two distances between the lamp and the panel: 500 mm and 1000 mm. With the FEL lamp distances were 500 mm, 707 mm and 1000 mm. Each lamp–distance combination produces different irradiance level at the panel. With each combination the FPI camera was used to acquire images with four different exposure times. These were 10 ms, 20 ms, 30 ms, and 50 ms for the Polaron lamp, and 10 ms, 15 ms, 20 ms, and 25 ms for the FEL lamp (Table 1). Each FPI camera acquisition consisted of 5 images with the optics covered to get dark image reading, followed by 10 images from the illuminated panel, and finally 5 more dark images. The uncertainty of the irradiance outputs were ±1.3% for the FEL lamp and ±1.0% for the Polaron lamp. Uncertainty of the panel reflectance factor was ±0.0045, ±0.0230, and ±0.0355 for the wavelength ranges of 490–800 nm, 810–870 nm, and 880–930 nm, respectively.

An inverse square law relationship scaling factor can be applied to get the Polaron lamp irradiance for the 1000 mm distance from the calibrated 500 mm distance. So $E\left(1000\;mm\right)=\frac{1}{4}E\left(500\;mm\right)$ for the Polaron. For the FEL lamp, there is a small offset *d* from the measurement plane that has to be taken into account at distances 707 mm and 1000 mm. The spectral irradiance *E*(*x*) of the lamp at distance *x* from the panel can be calculated using the absolute spectral irradiance of the source E(D) measured at the distance D with an offset from the measurement plane d:

(2) $$E\left(x\right)=\;E\left(D\right)*{\left[\left(D+d\right)/\left(x+d\right)\right]}^{2},$$

This is applied at each wavelength. With the offset value of d = 24.7 mm given by NPL, the irradiance scaling factors for FEL lamp distances 707 mm and 1000 mm are 0.514228 and 0.262198 respectively.

Irradiance at the panel E is converted to reflected radiance L using the following equation:

(3) $$L=\frac{1}{\pi }RE,$$

where R is the bidirectional reflectance factor of the panel. Finally, this radiance L was integrated to match FPI camera bands using accurate spectral response functions (Section 2.3.1).

**Figure 4:**
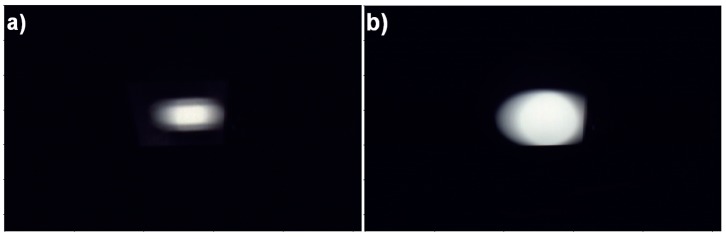
Example FPI images of the reflectance panel illuminated with (**a**) Polaron and (**b**) FEL lamp, 500 mm lamp distance. Only the centre part of the illuminated area was used for calibration.

FPI images of reflectance panel illuminated with the Polaron and FEL lamps from 500 mm lamp distance are shown in Figure 4 as an example.

The following processing procedure was performed for all radiance measurement setups to the FPI pseudoradiance images (Table 1). 10 FPI images were acquired from illuminated panel, followed by 5 dark images and each PFI image consists of 36 spectral bands. From each individual panel image, a rectangle of size 16 × 24 pixels for Polaron, and 30 × 30 pixels for FEL, covering the centre part of the panel was cropped and for each band these 10 cropped images were averaged to get the pseudoradiance panel image. Same rectangle was used for each individual dark image taken after the panel images, and these 5 cropped images were averaged to get the dark image. This dark image was subtracted from the panel image to get the pseudoradiances for each setup. Next the image was spatially averaged to get the pseudoradiance values per band for each setup. The final step was to normalize the pseudoradiance values by dividing with the integration time in milliseconds. This averaged and normalized radiance data per setup *L_Man_* in W/(m² sr nm), the radiance by sensor manufacturer, was used as starting point for further analysis of absolute radiance.

To calculate traceable radiances *L_New_* from the FPI camera radiances, a linear calibration model was used for each band using the NPL radiance data:

(4) $$L_{NPL\_ref}=a\times L_{Man}+b,$$

where a is multiplicative gain factor to compensate errors in absolute calibration and b is a linear term in radiance units to compensate differences in radiance levels. Band-wise parameters a and b were solved using least squares method. The reference radiance from NPL were used as $L_{NPL\_ref}$, and radiances measured from the panel FPI images were used as $L_{Man}$. New traceable radiance values *L_New_* are finally calculated with Equation (4) using band-wise parameters a and b and FPI camera radiances based on manufacturers calibration $L_{Man}$.

#### 2.3.3. Sensor Linearity Evaluation

Linearity of the sensor response to various radiance levels was evaluated using five radiance levels of Polaron and FEL lamps. 10 ms integration time was the only one commonly used with all five radiance levels. Radiances provided by NPL and integrated to FPI sensor response were used as reference and compared to adjusted sensor radiance *L_New_* based on Equation (4). Linear model was fit between the radiances and *R*^2^ values were calculated independently for each band.

#### 2.3.4. Evaluation of FPI Camera Stability

The FPI camera sensor is not temperature stabilized and therefore is susceptible to changes in external temperature and heating caused by internal circuitry. The magnitude of this effect was tested by setting the FPI camera to acquire images of a reference panel illuminated by a stabilized quartz tungsten halogen lamp. The ASD was also used to verify the stability of illumination. Both the lamp and ASD were switched on and left to stabilize for approximately an hour. Then dark images were acquired with the FPI camera, which was then set to acquire images with 10 s intervals, and left to run for 30 min. The external temperature of the laboratory was kept stable during the measurements.

Second test to evaluate the quality of the sensor manufacturer’s calibration was performed by comparing the radiances measured by the FPI camera and the ASD in a laboratory conditions at the FGI. Three different reference reflectance panels (named BC, GC and GP) and a maple leaf were illuminated using by a stabilized quartz tungsten halogen lamp, and the radiance of each target was measured with both FPI camera and ASD from 1 m distance. Each reference reflectance panel is sized 1 m × 1 m and their reflectance factors at 540 nm are 0.03, 0.09 and 0.53 for BC, GC and GP respectively [4]. 3° fore optics was used with ASD, and a 7 × 7 pixel, about 2 × 2 cm^2^, area of the leaf was measured from the FPI image.

### 2.4. UAV Data Capture and Data Processing Procedure

Feasibility of the proposed system was evaluated using datasets captured in 30 June, 2015 in the Wytham Woods test site in Oxford (UK, 51°46′33″ N, −1°20′12″ E). The airborne data capture was carried out using a hexacopter UAV with Tarot 960 foldable frame and Pixhawk autopilot. The FPI camera was fixed to look directly downwards. The OO spectrometer was on-board the UAV with cosine collector optics to collect the irradiance above canopy. The optics was mounted on top of the UAV to a stabilizing gimbal that was intended to keep the cosine collector pointing directly towards zenith. Additional components of the system included an on-board GPS (RasPiGNSS, NV08C-CSM, Cambridge, UK) that was used for collecting camera position trajectory for georeferencing purposes. A Raspberry Pi2 on-board computer was used to collect timing data for all devices, log the GPS trajectory and to save the OO spectra. A RGB camera (Samsung NX 300, Seoul, Korea) was also on-board during flights capturing high spatial resolution images that were used in the geometric processing. The ASD Field Spec Pro spectrometer was used to capture irradiance on the ground level during the flights.

**Figure 5:**
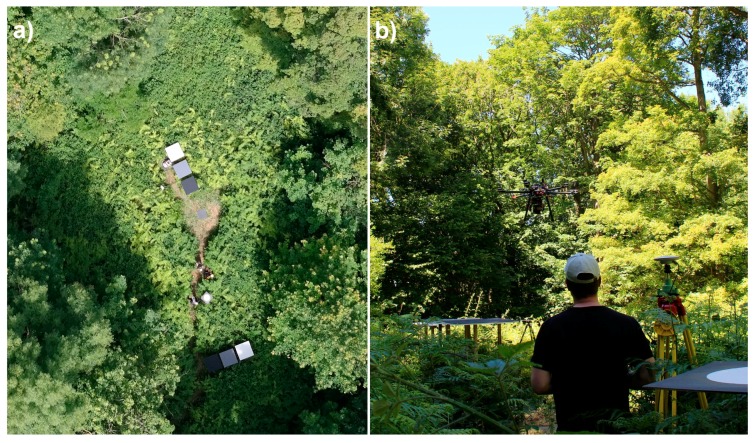
(**a**) An aerial image of the take-off and landing site. The reference panels BC, GC and GP in direct sunlight and in shadow are visible in image. (**b**) An image of the UAV landing site. The proximity of trees to the reference panels is evident in this image.

Six reference reflectance targets were installed in a small opening in the test area (Figure 5). A set of three panels (BC, GC and GP) were positioned in direct sunlight and another set in the shadow of the canopy. The reflectance factors of the radiometric reference targets were measured in field using the ASD spectrometer with 18-degree optics, by referencing to a white Spectralon reference panel. The spectra were sampled for the spectral bands of the FPI images.

The flight campaign was carried out in June 30, 2015. The time of data capture was 09:46 to 10:04 (GPS-time) in the morning, and the sun elevation and azimuth were 51.6° and 125.2°, respectively. The weather conditions were cloud free and windless. We captured an image block with 10 parallel flight lines aligned in the North-South direction. The nominal flight height was 90 m and for the approximate tree height of 20 m, the distance to the object was about 70 m; the resulting Ground Sample Distance (GSD) was about 9 cm at ground level and about 7 cm at treetop. The flight speed was 3.8 m/s and the exposure time was 5 ms. These settings provided the forward and side overlaps at the tree top level of 71% and 64% for the FPI camera, and 90% and 75% for the RGB camera, respectively. The FPI image data was pre-processed using the manufacturer’s software (Section 2.2.1).

Agisoft Photoscan Professional software (Agisoft LLC, St. Petersburg, Russia) was used in georeferencing to determine the image orientations and DSMs. In order to transform the image orientations to the WGS84 coordinate system, 6 signalised ground control points and GPS-trajectory of the flight were used. The outputs of the process were the image exterior orientations and the camera calibrations in the object coordinate system. In the case of the FPI camera, orientations of one reference band were determined in the block adjustment processing and the remaining FPI bands were optimized to the reference band. Dense point clouds with a 10 cm point density were generated using the Samsung NX 300 RGB images captured during the same flight. The geometric processing method used for the dataset is described in detail by Honkavaara et al. [31].

Due to poor performance of on-board stabilization of OO optics, the OO irradiance was unusable alone. To correct this, a procedure was developed to correct the level of irradiance measurement by fusing the ground based and on board irradiance measurements. A level shifting correction factor was calculated for the on-board irradiance data using the stable ground irradiance data from ASD. However, the proximity of the tree canopies caused significant effects to the irradiance spectrum, especially in the NIR, green, and blue spectral ranges. The effect in NIR and green spectral ranges is due to tree canopies reflecting light to the sensor, and in blue due to canopies blocking diffuse light from the blue sky from reaching the irradiance sensor. Red channel (600–700 nm) is least affected by the proximity of the trees. Corrected OO irradiance *OO_cor_* was calculated as follows:

(5) $$OO_{cor}=\frac{ASD_{red}}{OO_{red}}OO_{orig},$$

where *ASD_red_* and *OO_red_* are averaged ASD and OO irradiances on 600–700 nm wavelength range and *OO_orig_* is the original OO irradiance spectra.

Reflectance mosaics were then calculated with 20 cm GSD. The image orientations and DSM were used for the geometric transformation. The reflectance were calculated from radiance images and corrected *OO_cor_* irradiances using Equation (1). Both the radiance images based on the manufacturer calibration and the NPL calibration were used.

## 3. Results

### 3.1. Preliminary Study of FPI Camera Stability

**Figure 6:**
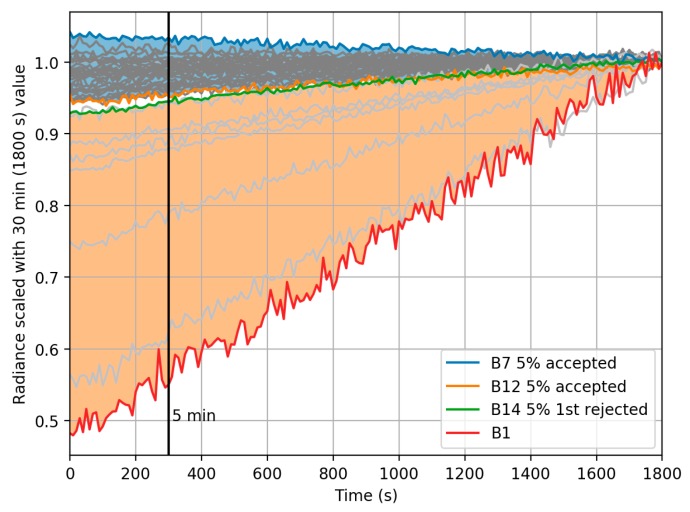
Time dependency of FPI camera radiance during 30 min. All data scaled with radiance at 30 min. Areas for accepted and rejected bands with 5% criteria between 5 min and 30 min are shown blue (accepted) and orange (rejected). Bands 7 and 12 are still accepted, bands 14 and 1 show the deviation of rejected bands.

Radiometric stability of the FPI camera response was assessed at the FGI measurement laboratory in a separate setup by simulating typical drone campaign. In typical operating procedure the camera is turned on for 5 min to warm before campaign, and the campaign time extends to 30 min at maximum. Relative FPI radiance difference in the time range of 5 min to 30 min was calculated, and if the difference was greater than 5%, the band was declared unstable (Figure 6). Based on this criteria, the following eight bands were declared unstable: 1, 2, 3, 4, 10, 11, 13 and 14. These bands were removed from the further analysis.

The sensor has two main sources of potential spectral calibration uncertainty: variation in band centre wavelength between image centre and edges (smile effect), and temperature dependency. Smile effect is well known and will be evaluated in the most accurate spectral analyses and temperature compensation has been implemented to the sensor to mitigate the effect of temperature.

**Figure 7:**
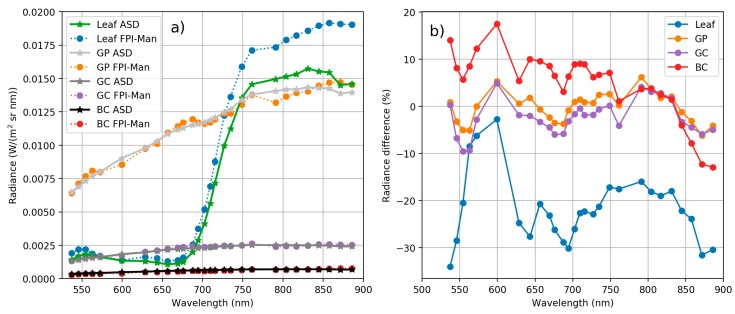
(**a**) Radiance spectra of reference panels BC, GC, GP and leaf measured with FPI camera based on manufacturer’s calibration (FPI-Man) and measured with ASD spectrometer. ASD spectra has been integrated to match FPI camera spectral responses. (**b**) Percentage difference between ASD and FPI *L_man_* radiance. Stable FPI bands only.

The quality of the FPI camera manufacturer’s calibration and sensor’s capability to produce realistic radiance values was evaluated at FGI laboratory by comparing radiance values from FPI camera based on sensor manufacturer’s calibration to ASD radiance over four different targets. The manufacturer’s calibration is based on the same ASD spectrometer that was used in the comparison. Radiance spectra of three different reference panels, BC, GC and GP, and spectra of leaf are shown in Figure 7.

ASD spectra has been integrated to match FPI camera spectral responses, see Section 2.3.1. Radiance spectra of reference panel matches well between ASD and FPI sensor. There is greater difference in radiance magnitude in leaf spectra at wavelengths over 750 nm. This can be due to different viewing geometry between ASD and FPI. ASD spectra were measured with 8° foreoptics, FPI spectra were averaged from a small area of interest from the centre of the image emulating the narrow ASD view angle. It is possible that FPI and ASD spectra are from different locations of the leaf. Radiance difference between ASD and FPI in percentages was mostly less than ±5% for the grey panels, less than ±10% for the black panel and between −5 and −30% for the leaf target. These results confirm that FPI camera provided consistent radiance spectra with respect to the ASD.

### 3.2. Sensor Radiometric Laboratory Calibration at NPL

#### 3.2.1. Spectral Calibration

**Figure 8:**
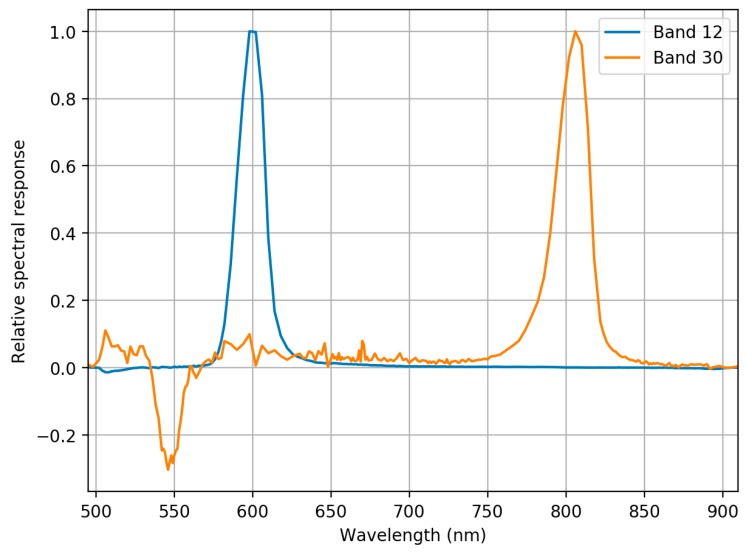
Example of full spectral response curves for bands 12 ($\lambda_{0}$ = 599.24 nm, FWHM = 19.82 nm) and 30 ($\lambda_{0}$ = 804.14 nm, FWHM = 25.13 nm). Curves are scaled so that the maximum value is 1.

**Figure 9:**
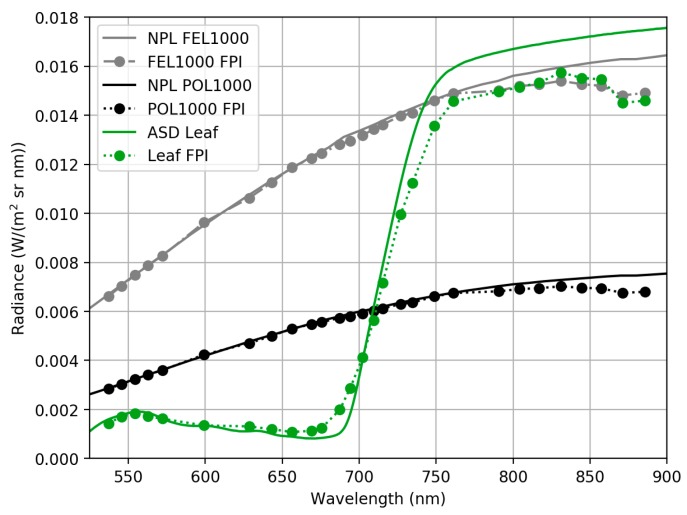
Original radiance spectra and same spectra integrated with FPI spectral responses. NPL FEL1000 and NPL POL1000: reference spectra provided by NPL; FEL1000 FPI and POL1000 FPI: corresponding spectra sampled using the FPI camera spectral responses; ASD Leaf: leaf radiance spectra measured using the ASD at the FGI’s laboratory; Leaf FPI: the ASD spectra sampled using the FPI-camera spectral responses. Leaf FPI is same as Leaf ASD line in Figure 7a.

Spectral responses for each band were measured at the NPL laboratory. Spectral responses for bands 10 and 30 are shown in Figure 8 and all bands in Appendix A. Many of the FPI 2012b camera bands have side peaks in spectral response, which impact the spectral measurements. Especially bands 25–36 have strong negative side peaks at 500–600 nm wavelength range. The negative values were due to the calibration coefficients from the manufacturer calibration that were applied to the images. It is possible to remove part of the leaking light by correction approach, but this was not developed for the sensor used in this study. Even when ignoring the side peaks, channel spectral response parameters (centre wavelength and FWHM) based on measurements at NPL differed significantly from the parameters of the manufacturer calibration; differences of the peak centre wavelengths ($\lambda_{0}$) were 5–10 nm and differences of FWHMs were also approximately 5–10 nm. The spectral calibration showed that the spectral response curves of individual FPI bands differed clearly from Gaussian shapes. This meant that we needed to integrate reference radiance using correct spectral response functions. The effect of FPI spectral response functions is demonstrated by showing original radiance spectra and integrated with FPI spectral response functions in Figure 9. Side peaks clearly affect to the shape of the radiance spectra by dampening its amplitude between 500 and 550 nm and at wavelengths above 750 nm, and also affecting to the slope of the red-edge.

#### 3.2.2. Radiance Calibration

**Figure 10:**
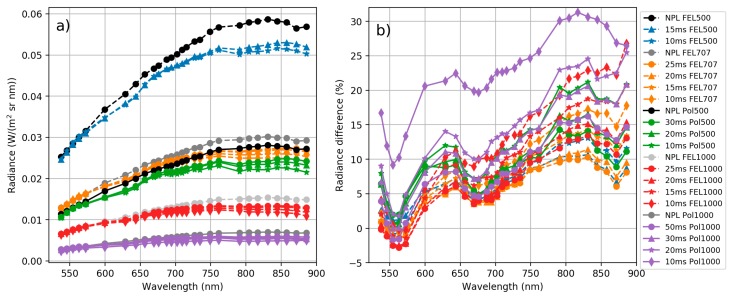
(**a**) Panel radiances based on manufacturer’s calibration. All FPI camera integration times, both lamps and all lamp distances, and NPL reference radiance for all lamp distances. (**b**) Manufacturer’s radiance *L_Man_* percentage difference to NPL reference radiance *L_NPL_ref_*. Values are given at centre wavelengths of the FPI camera channels.

Figure 10 compares the panel radiances from NPL reference *L_NPL_ref_* and from the FPI camera with manufacturer’s calibration *L_Man_* for each measurement setup. As can be seen from Figure 10, there are discrepancies both in spectral shape and absolute value between the FPI camera manufacturer’s radiances and reference radiances. The discrepancies were higher at the NIR than at the visible spectral range and reached the level of 30% at largest.

**Figure 11:**
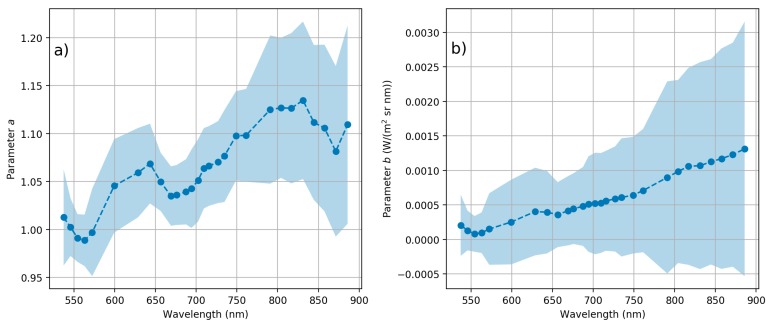
Band wise linear model parameters (**a**) *a* and (**b**) *b* to adjust *L_man_* radiance using Equation (6). Parameters solved using 11 data sets. Light blue colour indicates 95% confidence interval for parameters from least squares calculations.

**Figure 12:**
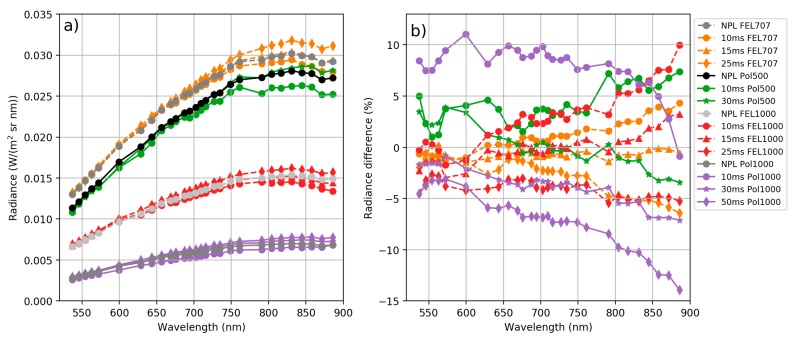
New adjusted radiance spectra *L_New_* (**a**) and percentage difference to NPL reference *L_NPL_ref_* (**b**) for 11 data sets used in the calculation of the calibration model.

**Figure 13:**
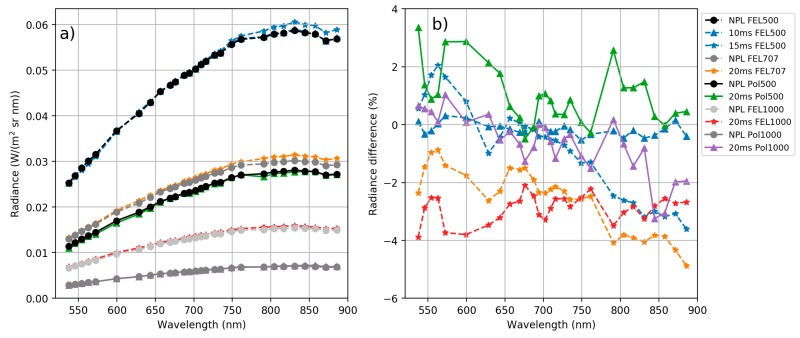
New adjusted radiance spectra *L_New_* (**a**) and percentage difference to NPL reference *L_NPL_ref_* (**b**) for six independent data sets.

Out of 17 data sets (Table 1), 11 were used to solve linear model per band *a* and *b* parameters (Equation (6)), and six data sets were left as independent test cases. Solved per band parameters *a* and *b* with 95% confidence intervals are shown in Figure 11. New adjusted radiance spectra of all cases *L_New_*, reference radiance *L_NPL_ref_* and absolute difference to reference radiance are shown in Figure 12 (11 data sets used in calculation of the calibration model) and Figure 13 (six independent data sets). When comparing the new adjusted radiance to the independent reference, differences were smaller than 4%, and in most cases <3%.

#### 3.2.3. Sensor Linearity Evaluation

**Figure 14:**
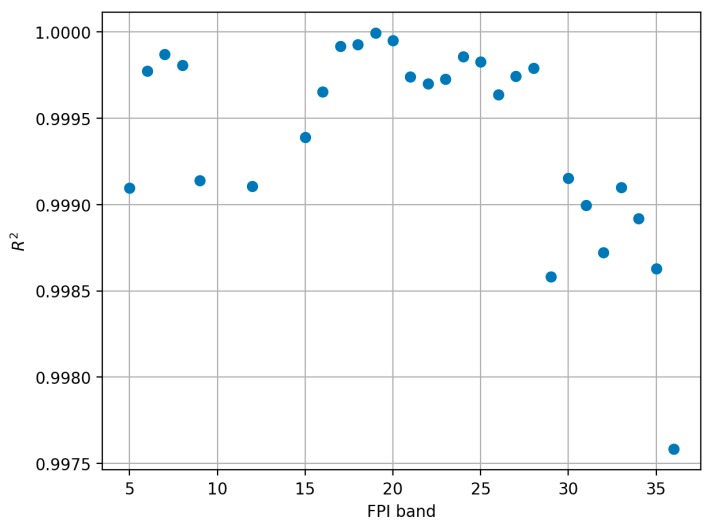
*R*^2^ values for linear fit between adjusted sensor radiance (10 ms sensor integration times) and NPL reference radiance. Stable bands only.

**Figure 15:**
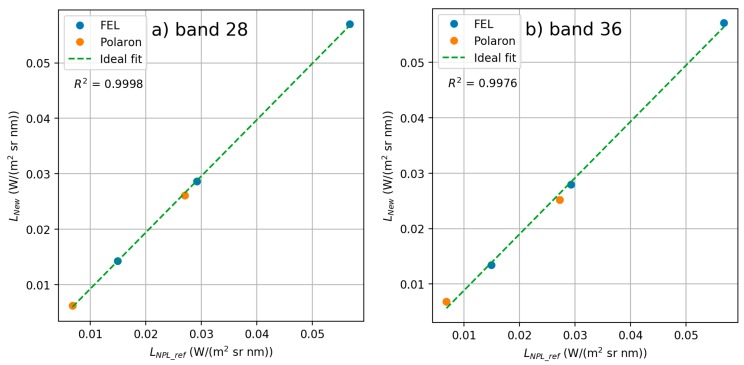
Linearity plots for (**a**) band 28 ($\lambda_{0}$ = 761.2 nm) and (**b**) band 36 ($\lambda_{0}$ = 885.9 nm).

Radiometric response of all stable bands of the FPI sensor proved to be linear with average *R*² of 0.9994. *R*^2^ of best bands (19 and 20) was 1.0000, and worst two were bands 36 and 29 with *R*² of 0.9976 and 0.9985 respectively (Figure 14). Examples of linearity plots for bands 28 and 36 are shown in Figure 15.

### 3.3. Drone Campaigns

#### 3.3.1. Processing of Ocean Optics Downwelling Irradiance Data

**Figure 16:**
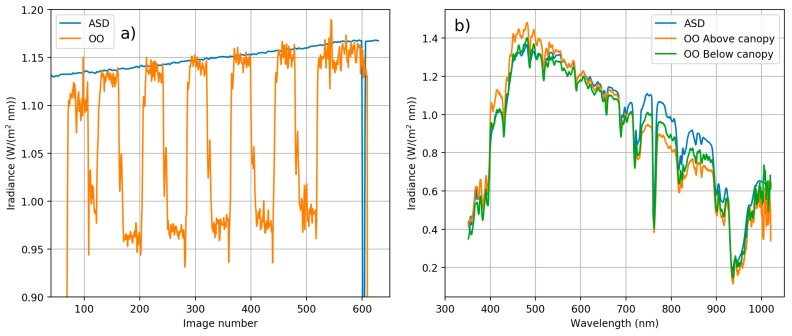
(**a**) Mean value of 600–700 nm irradiance spectra of the ASD and Ocean Optics (OO) for each FPI image acquisition time during the campaign. Irradiance increases toward the end of the flight due to rising solar angle. The steps in OO data are caused by UAV flying in different directions and thus the UAV and irradiance sensor tilting. (**b**) Effect of tree canopy to the irradiance spectrum. ASD is affected by the proximity of the tree canopies compared to the OO which is above canopy.

Important problem with the UAV based irradiance measurement is that the platform is not stable, and can be tilted to any direction, depending mainly on the motion of the UAV and wind direction. Unfortunately the gimbal of the OO did not sufficiently stabilize the cosine collector optics and the impact of different directions of the flight lines could be observed (Figure 16a). The correction method described in Section 2.4 was applied to the OO data to correct this. The surrounding vegetation affected to the irradiance spectra collected with ASD. Figure 16b compares ASD irradiance measured on ground and OO irradiance measured on board drone below and above canopy level. Both ASD and OO irradiance spectra below canopy have higher irradiance levels on red to near infrared wavelength ranges where vegetation has high reflectance.

**Figure 17:**
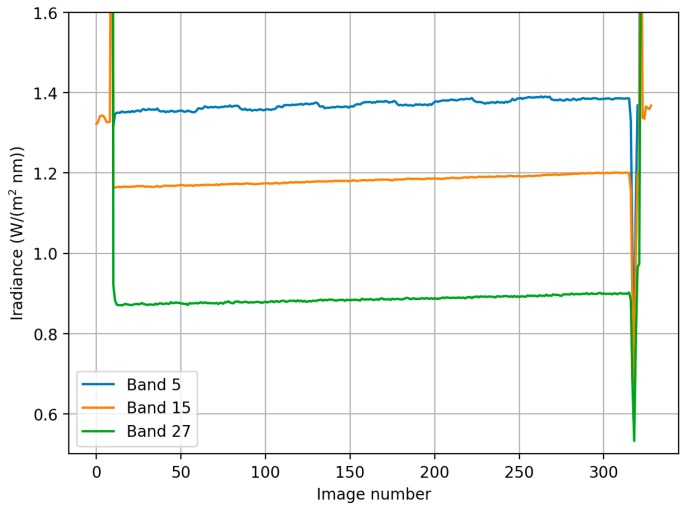
Corrected *OO_cor_* irradiance during the flight for blue (band 5, $\lambda_{0}$ = 537.2 nm), orange (band 15, $\lambda_{0}$ = 628.6 nm) and NIR (band 27, $\lambda_{0}$ = 748.8 nm) range bands.

The irradiance recordings during the entire flight were rising slightly due to rising of the sun elevation (Figure 17). The irradiance correction was of good quality excluding some undulations in the green band, which was due to some minor inaccuracies in the tilt correction.

#### 3.3.2. Reflectance Data Sets

**Figure 18:**
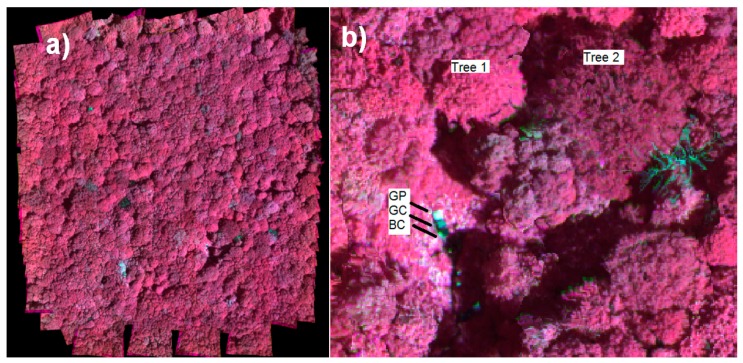
(**a**) False-colour reflectance mosaic of vertical flight f1 with FPI green, red and NIR range bands; (**b**) Part of the point cloud coloured with the spectral data, including locations of reference panels and measured trees.

We used both the image radiances based on the manufacturer calibration (*L_man_*) and those based on the traceable calibration at NPL (*L_New_*). The irradiance values were referenced to the ASD as described in Section 2.2.2. Orthophoto mosaics were calculated using the irradiance-based method. An example of an image mosaic is shown in Figure 18a. Also coloured point clouds were calculated as shown in Figure 18b.

**Figure 19:**
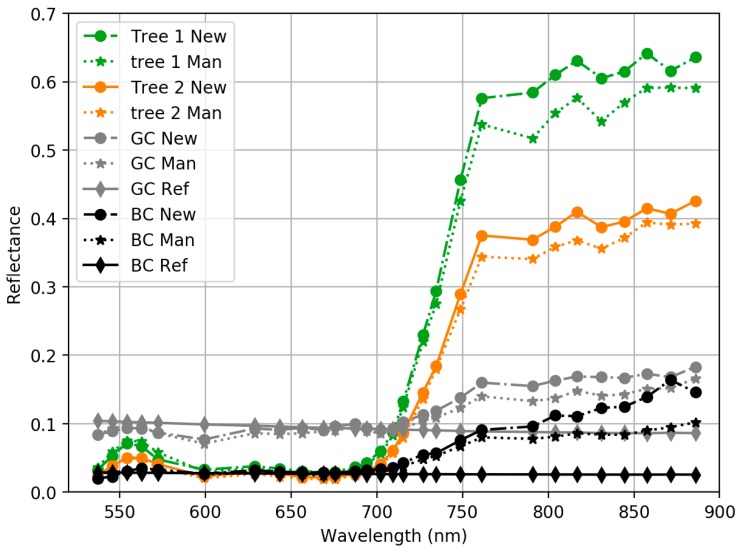
Reflectance spectra of sample targets based on original manufacturer calibration (**Man**) and the SI-traceable calibration at NPL (**New**). Also reference reflectance spectra measured at FGI laboratory for targets BC and GC are shown (**Ref**).

We used the reference panels BC and GC with nominal reflectance of 0.03 and 0.09 to evaluate the radiometric quality of calibrated image mosaics (Figure 19); brightest panel GP was excluded from analysis because it was saturated in many bands. In addition, reflectance spectra of two trees were measured from image mosaics. We used only the stable bands in this analysis (Section 3.1). The impact of surrounding vegetation could be observed in the panel spectra. In the NIR range, the background radiance due to the vegetation raised the level of incident irradiance and this could be seen in the panels’ reflectance spectra as increasing reflectance values. In the green and red spectral ranges, the surrounding trees were blocking the diffuse light that reduced the incident irradiance; this could be observed as reduced reflectance level of the panels in this range. In the NIR range, the panel reflectance collected from the hyperspectral image mosaic exceeded many times the reference values; in the green and red ranges they were closer to the reference spectra; at wavelengths less than 680 nm, the RMSEs were approximately 7.6% and 7.7% for the manufacturers’ and NPL calibration, respectively. Overall, the reflectance measured from the mosaic with NPL calibration appeared slightly higher than expected level; the values with the manufacturer calibration were closer to expected level. 

Smaller differences with the manufacturer calibration were due to fact that in manufacturer’s calibration both OO irradiance sensor and FPI camera were calibrated with respect to the same ASD spectrometer. The proposed direct reflectance measurement method can be considered as highly promising; in order to improve the reliability and decrease the uncertainty, further developments of sensor calibration and direct irradiance measurements are necessary.

## 4. Discussion

The empirical line method is a useful approach in many practical applications to transform the image intensity values to reflectance because the UAVs are mostly operated locally and it is easy to install reflectance panels in the area of interest [16,32]. However, due to challenges to obtain accurate calibration by reflectance panels in many situations, the direct reflectance measurement based on calibrated imaging spectrometer recordings and incident irradiance recordings on board UAV are highly attractive. When calculating the reflectance as the ratio of the incident and reflected radiance based on the imager and the irradiance spectrometer, the radiometric calibration of the two instruments becomes crucial task.

The 2D spectrometer used in this study is based on the tuneable FPI. This represents novel miniaturized technology which is highly attractive for the UAV use because it allows a possibility to carry out hyperspectral 3D reflectance and radiance measurements of the objects [1,2,3]. The 2D format images provide complete coverage over the object of interest, giving a possibility for highly detailed 3D measurements, without any gaps in the data; the point and pushbroom spectrometers might have challenges to provide continuous data. On the other hand, rigorous calibration of the 2D-format sensor can be more challenging than that of pushbroom or point based spectrometers, in particular, due to their wide field of view and due to the complex imaging principles. Previous studies have studied temporal performance [1,8], vignetting calibration [1,8,16], and radiance calibration [16]. This was the first study assessing the radiance and spectral response of a 2D frame format camera.

The complete system calibration was carried out by the manufacturer using a non-traceable procedure; the radiance calibration was based on the ASD Field Spec Pro spectrometer of the FGI. The FPI camera’s spectral response and radiance factor for one selected exposure setting were calibrated using the SI-traceable procedure at the NPL. The spectral calibration showed that many of the bands had side peaks caused by the secondary interference effects of the FPI; this effect impacts the spectral quality but probably could be compensated for when the spectral response of the camera is known; this aspect has to be studied further. The effect of side peaks was strongest on NIR-range channels and it also affected to the slope of red-edge. The impurity of the spectral bands, if not cleaned, limits the usability of the sensor in collection of object reflectance data for libraries. If applying spectral libraries in data analysis, the impurity can be accounted for by utilizing the spectral responses of bands. In applications where the classifiers or estimators are trained with in situ observation data the impurity is not in most cases critical but can cause some reduction of accuracy. It is also possible to seek an air gap sequence for the sensor that results in cleaner bands, but in this study we were using the most common air gap sequence used in our practical applications. In the latest commercial version of the Rikola FPI camera by Senop Ltd. (Oulu, Finland), the FPI side peaks are minimized by dividing the spectral data to two different CMOS-sensors without any Bayer pattern on them.

The FPI camera radiances based on the manufacturer calibration had 0–30% differences to the SI-traceable radiances at NPL, which indicated that the manufacturer calibration, traceable to the ASD spectrometer, was biased. Analysis using the independent SI-traceable references showed that after calibration at NPL, the FPI radiances differed ±4% from the reference measurements in different bands. 

Our characterization at the laboratory showed that the radiometric response of some of the bands drifted with time. To manage this effect, temperature information of the sensor would be necessary. For the camera used in this study, this information was not available thus we are using the bands that are least impacted with the sensor warming. The sensor also has quite remarkable smile-effect, which was not calibrated in this study because the calibration setup enabled only the calibration of the centre part of the camera. The calibration was performed at the focus distance thus the calibration target covered only a small part of the sensor field of view. The typical calibration procedure is to calibrate the imaging spectrometer cameras by taking a picture out-of-focus directly at the output port of the integrating sphere; impact of this should be studied in further studies.

Some previous studies have used irradiance spectrometers on board UAV in order to measure the incident irradiance and to transform the spectrometer radiances to reflectance, or compensate the illumination changes. The previous studies have discussed about challenges in correction of tilting of irradiance spectrometers on board UAVs [21,23,24]. This was also problem in our study; the gimbal that we used to compensate for the tilting of the spectrometer optics did not give acceptable results. We developed a procedure where we utilized the ground based spectrometer for compensating the impacts of tilting of the sensor. Despite of many challenges in the processing the direct reflectance measurement provided consistent reflectance measurements. In the future drone campaigns, our solution for stabilized irradiance measurements is to use FGI AIRS, FGI Aerial Image Reference System, that uses optical levelling to compensate tilting of the irradiance sensor [27]. FGI AIRS can be easily paired with any imaging sensor providing output signal of the image capture times.

Our proposed system for direct reflectance measurement from drone provides reflectance factors at sensor level, which is strictly speaking different than reflectance factor at object level i.e., on ground or at tree tops. But when the drone flying heights are typically between 50 m and 150 m above ground, the atmospheric disturbances between ground and drone can be considered minimal. In future studies, comprehensive evaluation of the reflectance accuracy and quality of the proposed system should be studied in field using various reference targets with different spectral shapes and brightness levels.

## 5. Conclusions

We presented the first study on direct reflectance measurement using a UAV-based calibrated imaging 2D hyperspectral camera and a calibrated irradiance spectrometer. The approach is extremely attractive, as it simplifies the field operations, and it is suitable for operations in varying illumination conditions, in densely vegetated areas (forests) and beyond line of sight. The system provided consistent reflectance measurements, indicating that further developments are highly relevant in order to develop the approach to fully operational level. The rigorous system calibration is a crucial step in this process. In this study, the imaging spectrometer was calibrated using a SI-traceable procedure at the National Physical Laboratory. Results showed inaccuracies in the camera spectral responses and indicated that the radiance calibration of the ASD spectrometer used as the reference in the manufacturer calibration was not consistent with the SI-traceable radiance at NPL. These findings highlight the importance of developing accurate and efficient calibration procedures for the novel, hyperspectral imaging sensors. As soon as the sensor calibration and irradiance measurement stabilization challenges are solved, highly efficient UAV based direct imaging spectroscopy techniques can be developed.

## Figures and Tables

**Table 1:** Different image data sets used in radiance calibration. Distance is lamp distance from the reference panel. Data marked normal font were used in calculating new conversion factors for radiance, data marked with bold were used as independent test cases for evaluating the radiance accuracy.

Lamp	Distance (mm)	Integration Times (ms)			
Polaron	500	10	**20**	30	-
	1000	10	**20**	30	50
FEL	500	**10**	**15**	-	-
	707	10	15	**20**	25
	1000	10	15	**20**	25

**Figure A1:**
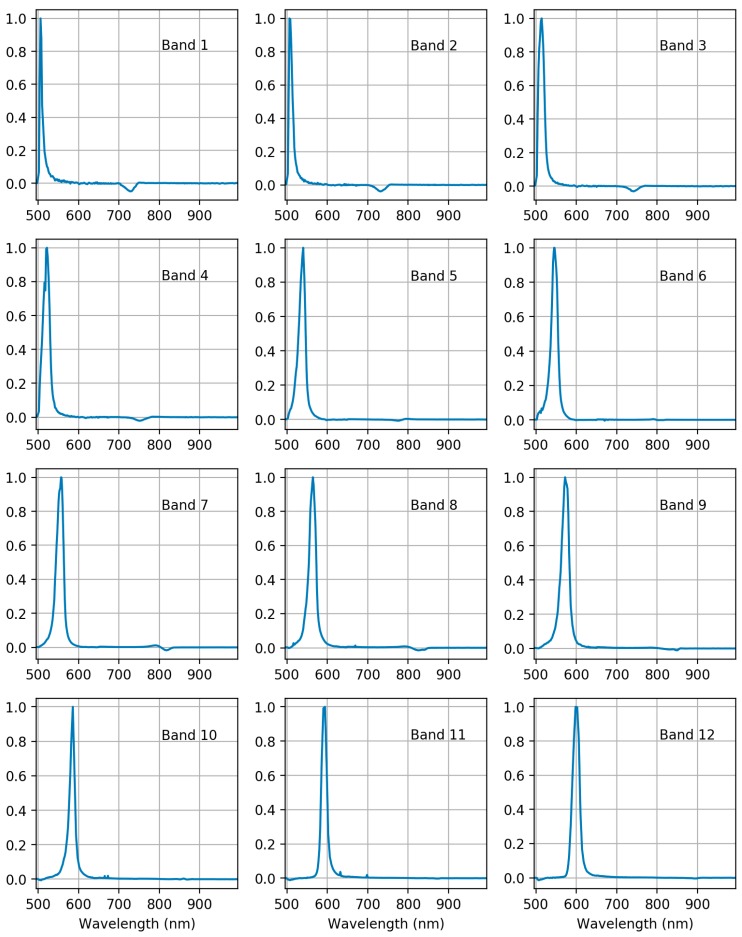
FPI spectral response curves for bands 1–12. Curves are scaled so that the maximum value is 1.

**Figure A2:**
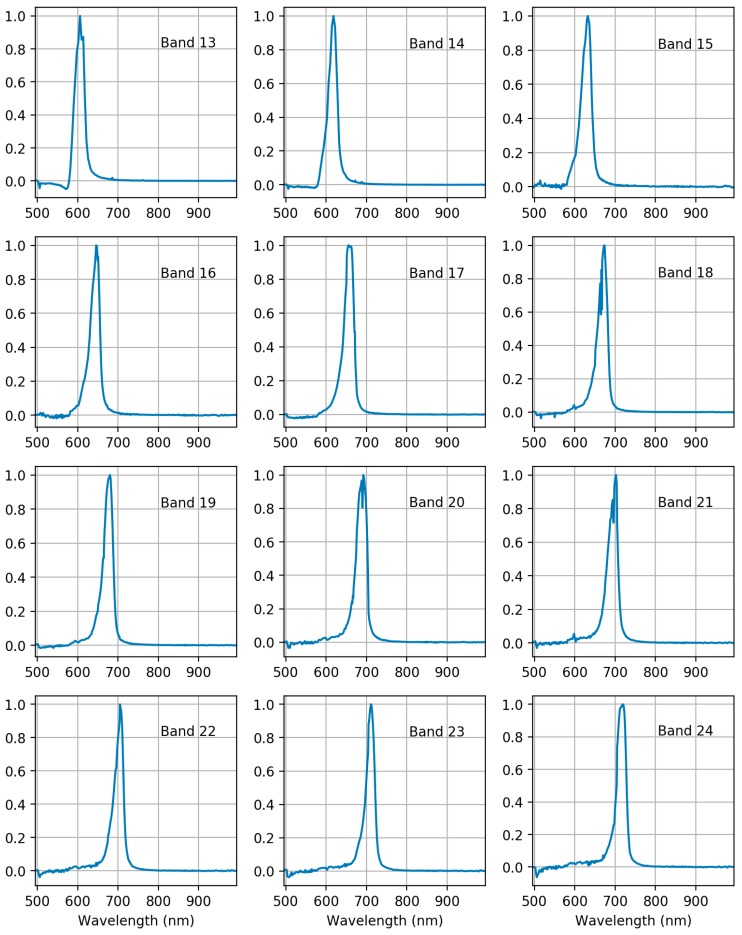
FPI spectral response curves for bands 13–24. Curves are scaled so that the maximum value is 1.

**Figure A3:**
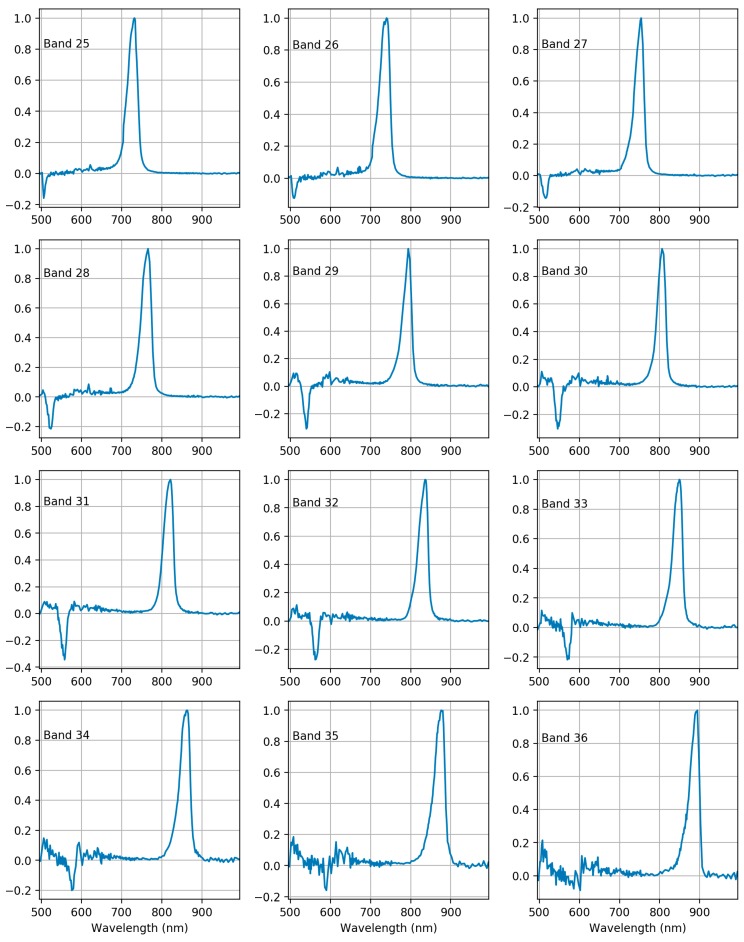
FPI spectral response curves for bands 25–36. Curves are scaled so that the maximum value is 1.

## Appendix A

Spectral response functions of all FPI camera bands are shown in Figure A1, Figure A2 and Figure A3.
